# Maximizing Foreign Body Detection by Ultrasound With the Water Bath Technique Coupled With the Focal Zone Advantage: A Technical Report

**DOI:** 10.7759/cureus.31577

**Published:** 2022-11-16

**Authors:** Samantha C Shelhoss, Chelsea M Burgin

**Affiliations:** 1 Emergency Medicine, Prisma Health, University of South Carolina School of Medicine, Greenville, USA

**Keywords:** focal zone, soft tissue infection, skin and soft tissue, focal zone on ultrasound, water bath ultrasound, bedside ultrasound, pocus (point of care ultrasound), retained foreign body, foreign body on ultrasound, superficial foreign body

## Abstract

Advancements in medical technology and clinician education have significantly improved ambulatory clinicians' access to bedside ultrasound. The presence of a foreign body can be identified on ultrasound as a hyperechoic disruption of soft tissue accompanied by posterior shadowing, reverberation artifact, and/or an anechoic halo. Although the literature discusses the utility of point-of-care ultrasound for foreign body identification and removal techniques, there is a gap in current literature advancing detection techniques for foreign bodies in small structures. This technical report highlights the role of the water bath technique while incorporating the focal zone to aid in the discovery of small objects in hands and feet.

## Introduction

Foreign bodies (FB) are common ailments that present to emergency, primary and urgent care centers. Detection of a large (>2 cm) superficial FB can be done by gross inspection and palpation; however, imaging such as X-ray, computed tomography (CT), magnetic resonance imaging (MRI), and ultrasound are often required for a small foreign body. Hands and feet are common areas for a FB to embed due to their constant contact with the environment.

In the last three decades, point-of-care-ultrasound (POCUS) has become a versatile instrument in the clinical toolbox across an increasing number of medical specialties. The literature spanning these specialties describes the role of POCUS or bedside ultrasound in a variety of applications, including biliary, blunt trauma, chest pain, critical care, dyspnea, hypotension, musculoskeletal, skin and soft tissue, as well as vision changes [1-3]. The ultrasound application for skin and soft tissue serves as a tool in discerning the presence or absence of an abscess or masquerader when the patient history and clinical examination are inconclusive. Skin and soft tissue ultrasound can expedite care to resolution when the concern of an implanted foreign body presents, as it can aid in detection and removal [4-7]. The smaller the FB, the more difficult it is to identify, which further supports the need to discuss methods to improve detection by ultrasound. 

The water bath technique submerges the body part of an interest in the water while hovering the probe above the skin to serve as a magnifier. In addition to the magnification, another advantage of a water bath compared to the gel technique is reducing pain/pressure compared to gel and direct contact with the probe. The water bath, coupled with the incorporation of the focal zone during image acquisition, will further advance success rates in superficial FB detection by ultrasound. The water bath technique was first discussed nearly two decades ago [8] and deserves revisiting as handheld devices continue to improve clinician access and may drive the standard of care in the near future for emergency, primary, and urgent care clinicians who evaluate patients with suspected FBs. The focal zone is a long-standing physics concept in ultrasound and determines the best two-point discrimination within an ultrasound image.

## Technical report

As with all POCUS applications, superficial FB detection by ultrasound requires knowledge and skill. In the hands of experienced ultrasound clinicians, FBs as small as 2.5 mm can be detected for removal [4]. Regardless of FB composition, organic or inorganic matter, most FBs appear hyperechoic on ultrasound, like cortical bone. Often FBs are accompanied by reverberation artifact, posterior shadowing, and/or a hypoechoic halo. The most frequent locations for a FB to embed are related to the high activity level of the body parts that contact the environment. Due to the fine motor skills of the fingers and hands, in conjunction with the constant necessity of feet making contact with the ground for ambulation - all contribute to the hands and feet being high-incidence areas for FB. The following guidelines include the water bath technique to aid in the advancement of a novice or experienced POCUS clinician in FB detection, specifically in hands and feet. 

Image acquisition

The needed equipment is a water basin or wide sink, room temperature or cool water (some ultrasound probes are sensitive to overheating), and an ultrasound device.

**Figure 1:**
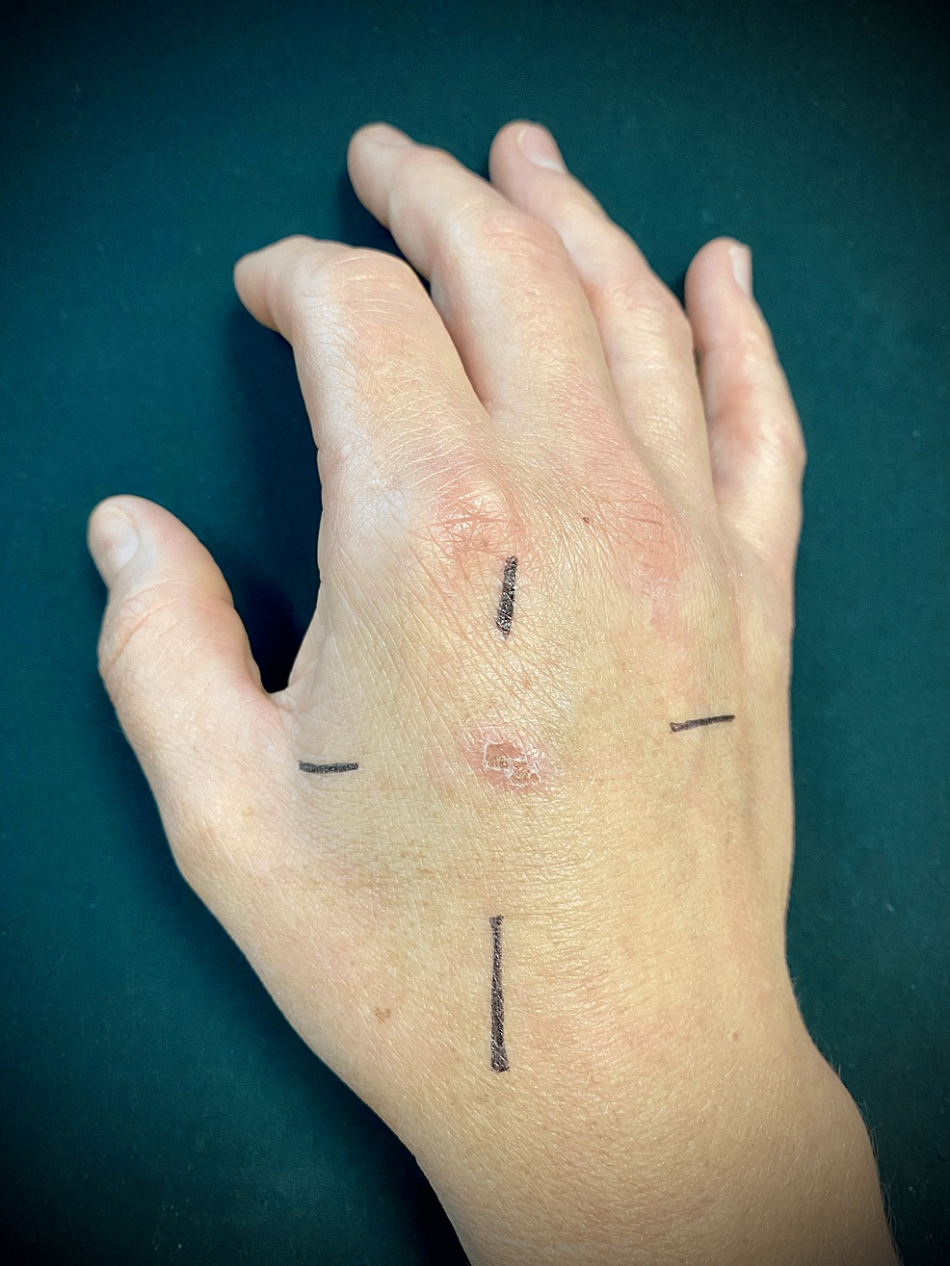
Skin preparation for foreign body detection

**Figure 2:**
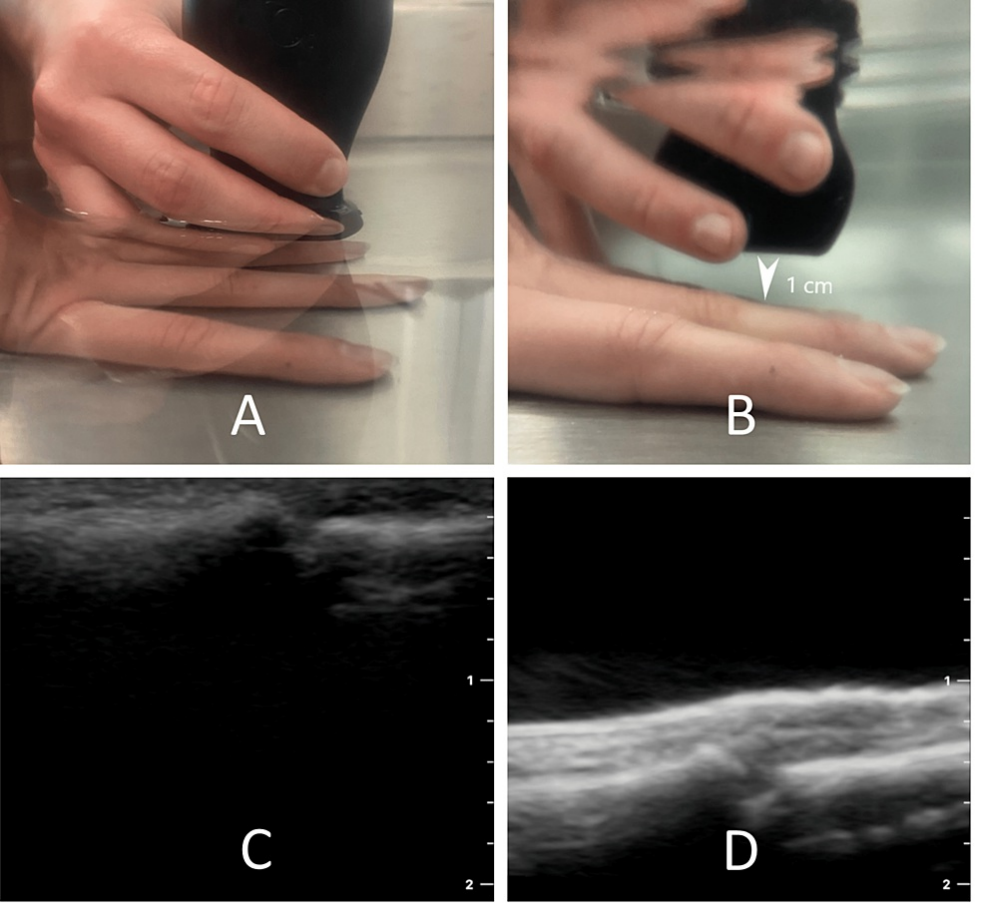
The water bath technique A) Above-water view of the patient undergoing ultrasound in a water bath. B) Underwater view highlighting the clinician's pinky finger acting as an anchor with 1 cm of water between the probe and the patient. C) The ultrasound image of the patient's finger with the probe in contact with the skin illustrates the difficulty of seeing the dermis clearly with the horizontal hyperechoic cortical bone centering the interphalangeal joint at the top of the image at 2 mm depth (gel view). D) The same finger by ultrasound in a water bath with 1 cm of water distance between the probe and skin surface, highlighting clarity in the top hyperechoic line as the dermis and the second hyperechoic line at 1.4 cm depth as the phalanges with interphalangeal joint resting right on top of the image "D" label.

1. Mark the area of interest with a pen, as illustrated in Figure 1; extend the markings beyond the footprint of the probe to assist with centering when underwater, as in the image below.

2. Submerge the suspecting body part in 6-8 cm of water, as displayed in Figure 2A. The entry wound or region of greatest concern should be nearest the water surface, facing the probe's scan surface.

3. Using a linear transducer and/or superficial preset, hold the probe like a pencil using a thumb through ringer finger to maneuver the probe with the pinky finger acting as an anchor against the basin/sink or patient.

4. Hover the probe approximately 1 cm above the skin, as in Figure 2B, centered over the area of interest, to magnify the structures while increasing the water space between the area of interest and the probe to maximize its proximity to the focal zone. Please note the total screen depth may need to be reduced on the ultrasound device to contribute to the focal zone advantage.

5. Maintain the distance between the probe and skin while sweeping over the anatomic area of concern in multiple planes, starting in the long or short axis of the soft tissue. 

Note that, If a FB is not detected, rotate the probe 15-45 degrees to include oblique sweeps. The most reliable way to identify a FB is to sweep through the FB in its long axis, where it will appear the largest. Once identified, make a note of where the FB is nearest the surface of the skin and be cognizant of adjacent landmarks, such as vessels or joint spaces, that may complicate a removal procedure. FB removal is impossible in a water bath; as such, the water bath is a detection advantage only.

Image capture

During or after image acquisition, capture the study for the medical record with labeled sweeps in 4-6 second video clips, both in long and short axis relative to the FB. Consider a still image with a size of FB and depth measurements. With the color doppler feature turned on, capture a video clip highlighting adjacent vasculature.

Image interpretation and implementation

Make a note of the orientation of the FB relative to the surrounding tissue, whether parallel, perpendicular or oblique to adjacent connective tissue, specifically proximity to the skin surface and vasculature. When a FB is confirmed by ultrasound, shared medical decision-making is advised when determining the necessity of its removal. Pain and infection are indications for FB removal. Several key variables are involved when determining whether it is appropriate for a non-surgeon to pursue a FB removal. Considerations include but are not limited to the clinician's skillset and level of experience, access to necessary equipment and anesthesia, as well as resources to manage potential complications such as bleeding. Due to the unique nature of each FB scenario, the decision to remove versus the referral should be determined on a case-by-case basis. The brief Video 1 below summarizes a case of FB detection using the focal zone advantage via a water bath. 

**Video 1:** Water bath with the foreign body to achieve the focal zone advantage

Tips for Dynamic Ultrasound Guided FB Removal

A. Identify where the FB is most proximal to the skin surface and introduce local anesthesia in that region first.

B. When possible, use hydro dissection [7] through live ultrasound guidance following the needle tip and injecting anesthesia while advancing the needle towards the long axis view of the FB. The injected fluid appears anechoic and will dissect tissue planes creating an anechoic halo around the FB once in the correct tissue layer.

C. It is recommended to leave the needle in place just deep into the FB for use as a procedure guide. The probe can then be set down with two removal options: 1) incise the skin and cut down to the needle (which may elicit more bleeding), or 2) make an incision through the dermis and then dissect through the subcutaneous tissue towards the needle by spreading apart tissue with a hemostat or scissors. Due to capillary disruption, bleeding creates a nearly blind field, and removal is typically completed through instrument feel. Avoid placing the clinician's finger blindly into tissue when a sharp object is suspected.

D. After removal of a FB, if able (with respect to infection prevention), repeat ultrasound to ensure there is no visible retained foreign matter.

Infection prevention 

Infection prevention is an important consideration in every ultrasound encounter. It is paramount to utilize approved disinfection wipe(s) before and after each use and high-level disinfection when indicated [9]. If there is a potential for probe soiling through broken skin, apply an approved sterile probe cover prior to image acquisition. Caution is advised as the use of a routine disposable glove is unacceptable due to its permeability and lack of protection against small viruses such as HIV or hepatitis.

## Discussion

The incorporation of POCUS for FB detection in clinical practice is applicable to clinicians in ambulatory and hospital facilities and from prehospital care to nurse practitioners, physician assistants, as well as pediatric and adult physicians [10-12]. In general, there are four imaging modalities for the detection of FBs: X-ray, CT, MRI, and ultrasound. Vegetative or wooden FBs are notoriously difficult to identify by static imaging, particularly if the FB is small and without associated fluid or abscess [4,13]. In the presence of reoccurring or persistent infection or pain, MRI has been found to be superior to X-ray and CT [14] though its superiority to ultrasound is being challenged due to the dynamic process of bedside ultrasound adding unique value to this modality, in addition to its bedside capability by the treating clinician.

X-ray

Despite X-rays being the most consistently available imaging resource for inpatient and outpatient settings, the lack of sensitivity and specificity limits the efficacy of X-rays for the detection of many FBs. Between 1976 and 1982, an American study identified that up to 38% of FBs in hands were missed on initial presentation [15], as is the case with the radiolucent matter. Handheld ultrasound devices are introducing a wave of change as the convenience of this imaging opportunity can be as accessible as the clinician's pocket. An Asian study published in 2020 discussed the value of handheld ultrasound in search of FBs in the presence of chronic inflammation. In this study, the mean time between injury and FB identification was 10.5 months in the patients who recalled a time of injury; however, in 35% of the patients, nine out of 25 did not recall an injury [16]. The detection of a FB by ultrasound can expedite care, improve outcomes, and should be considered early, particularly in situations when a FB may be radiolucent and undetectable by X-ray [4,13,14,16].

CT

FB identification by CT scan compared to X-ray has a significantly higher detection rate, although CT is expensive, and the accompanying radiation and access to image acquisition limit CTs utility when compared to bedside ultrasound [4]. Additionally, CT is limited in its reliability in detecting plastic [14]. MRI, like ultrasound, has a higher sensitivity when compared to CT in ruling out FBs. 

MRI

MRI, though considered the current gold standard, is not an appropriate imaging modality in all scenarios. MRI may be hazardous to the individuals involved as well as the equipment during image acquisition in the presence of ferromagnetic or metallic matter [4,14,17]. In 2017 the National Institute of Health (NIH) published a peer-reviewed safety report providing insights into MRI limitations. The first limitation was access - at that time, there were only 30,000 MRIs globally. Secondly, identifying all ferromagnetic substances internally and externally on all individuals entering the higher-risk MRI zones can be difficult [17]. Thirdly, psychologic stress response(s) in individuals with claustrophobic tendencies can limit the tolerability of the imaging procedure(s) [18].

MRI is less available in the timely sensitive scenarios to primary and urgent care settings and is notably more expensive than other modalities. MRI has other unique limitations with respect to patient size and pre-existing instrumentation compared to ultrasound, specifically in terms of superficial FBs.

POCUS

Bedside ultrasound has three main advantages when compared to the above static imaging modalities for FB detection: timely access, dynamic imaging by the clinician, and potential guidance for removal of the FB, which can reduce local tissue damage. Reliable access is a goal to strive towards, as it requires leadership support, planning, training, and resources with sustainable infrastructure. These measures are achievable in emergency departments, primary and urgent care settings. Variables that can limit the efficacy of bedside ultrasound are its dependency on the skillset of the sonologist and access to charged, clean, safe, functioning handheld devices.

The Sonologist

A sonologist is a three-in-one clinician who incorporates POCUS into their clinical toolbox: 1) acquire: the sonographer or ultrasound technician, 2) interpret: the image interpreter (like a radiologist), and 3) act: the clinician who incorporates the former two into the treatment plan expediting care and/or improving outcomes.

The Focal Zone Advantage

**Figure 3:**
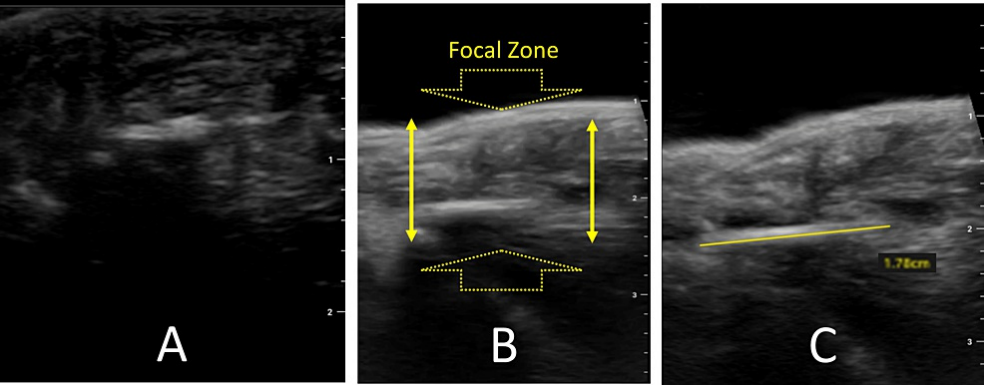
The focal zone advantage through a water bath A) Ultrasound probe on skin with gel and difficulty detecting the hyperechoic horizontal FB at 8 mm depth. B) Image of the same FB, slight oblique lay, in a water bath marking the focal zone. C) Total depth of the image reduced and probe adjusted, placing FB in true long axis, improving measurement accuracy. Note: posterior shadowing and some anechoic fluid create an early "halo sign" seen in images B and C yet indiscernible in image A.

For small structures, such as hands and feet, understanding one basic concept of ultrasound physics will help a sonologist improve success rates in the detection of small FBs. This is called the focal zone. The focal zone is the region within the ultrasound image where there is the most accurate two-point discrimination when the sound waves are most dense relative to each- other. The focal zone is dependent both on the ultrasound preset and depth; in highly articulate equipment, the focal zone may be manually adjusted. Using a high-frequency linear probe in a superficial preset lays the foundation for a sonologist to incorporate a water bath while utilizing the focal zone advantage in search of small FBs. Figure 3 marks the focal zone while comparing ultrasound images of a finger containing a wooden splinter, first using gel, then water bath views.

The Water Bath

The role of the water bath is twofold and can be crucial in the detection of small FBs in small structures. The first role of water is to be an ideal conductive medium. Water is low cost, readily available, and easy to volume adjust while preventing the need for contact between the probe and the patient's affected structure. The advantages of the water bath include the avoidance of pain and anxiety, as water replaces the need for contact between the probe and sensitive tissue. Once the superior aspect of the body part is submerged with 2-6 cm of water above it, the sonologist can maneuver the probe to bring the anatomic area of concern from the near or far zones into the focal zone prior to anchoring the probe hand with the pinky finger to stabilize for minute probe maneuvering. A disadvantage of the water bath is one of logistics; for example, a foot may not easily submerge to create an environment conducive to this technique. An alternative to the water bath can be a mountain of gel or a glove/bag filled with water to create a conductive medium space broad enough to facilitate the focal zone advantage.

The POCUS Advantage

The value of POCUS increases as it takes into consideration the patient history and physical exam while the clinician and patient interact before, during, and after bedside ultrasound with the potential of FB removal. The dynamic characteristics of bedside ultrasound make it a superior imaging modality. No X-ray, CT, or MRI pairs the patient and clinician in such a favorable manner.

An argument can be made for ultrasound-guided removal of a FB prior to surgical referral [19]. An ultrasound-guided approach on initial presentation is timely with the additional benefits of being less expensive, available at the bedside, and advances minimally invasive procedures while increasing the likelihood of removal. Scheduled surgical removal can entail a time delay between embedment and removal, FB migration, and more complex wound exploration with increased scar size. Ultrasound guidance via static or dynamic removal supports a small incision [19]. A recently published technical report highlights ultrasound in its pre-operative skin marking utility [20], as discussed above, interestingly, through the radiology department prior to the operating room. It is time for clinicians to strive towards becoming sonologists at the bedside using these ultrasound skills together (acquire, interpret and act) to increase successful identification and removal on initial presentation.

Limitations of bedside ultrasound in FB identification are important to acknowledge. Equipment access can be a barrier due to overall costs and infrastructure or as probes/devices may be unavailable due to infectious disease cleaning protocols. Prolonged turnaround times can be a challenge if high-level disinfection is required yet only available out of the clinical department. Other equipment constraints include hardware damage (cracked probe, frayed cord, etc.) or software failures. Although ultrasound is good at identifying organic and inorganic, radiolucent, or radiopaque materials, the size of a FB can limit its detectability. One study training military paramedics showed a drop of 18% in FB identification when the size of the FB dropped from 2 mm to 1 mm [9]. Different probes and software systems provide varying levels of image quality; a linear probe with high frequency is ideal. 

If the ultrasound equipment is clean, intact, and functioning, with the correct probe, and present, the limitations are more operator dependent and rely on the clinician's skillset in conjunction with patient tolerance. A patient will need to cooperate with the clinician in terms of their ability to remain still. The patient's habitus and access to a container to create the water bath may also be a challenge, as a large foot in a standard soak basin may not be feasible.

Operator limitation is the fact that the clinician will need to maintain a constant grasp of the probe as well as a finger anchor for steady scanning of the body part for FB. A focused limitation of the water bath itself, though it magnifies and eases identification, adds a modest level of complexity in terms of probe maneuvering and anchoring underwater and only has utility in searching for an FB before and after suspected complete removal, not for the removal itself. The severity of the FB presentation can also limit ultrasound utility; if the entry wound, initially thought of as superficial, was found to be accompanied by more severe injury such as arterial bleeding, the use of a water bath and bedside ultrasound may have less utility.

Complications from retained FB can include infection, reduced body part function, psychological stress, and pain [11,17]. As a result, unresolved injury relating to retained FB has been accompanied by a high level of medicolegal resource consumption [16]. Like all imaging modalities, there is a risk of missing a foreign body; thus, ongoing efforts need to be diligently exercised to educate a patient on next-step care plans. Keeping in mind the nature of human tissue with imbedded FBs, repeating dynamic ultrasound may improve FB identification and repeating POCUS after FB removal to ensure all components have been removed. An interstitial fluid halo or fibrous encapsulation may develop around the foreign matter; both of these physiologic responses to a FB would ease in its visualization by ultrasound. These considerations add levels of protection and value to the patient and treating clinician.

## Conclusions

POCUS is becoming the standard of care across an increasing number of clinical scenarios and specialties. Bedside ultrasound is an indisputable asset to the clinician when specific questions are present with the need for timely answers, as in the scenario of a potential FB. The skin and soft tissue application for ultrasound have a growing role in ambulatory and hospital settings, particularly with equipment and resource advancements such as hand-held ultrasound devices. 

The water bath technique for the ultrasound of the hands and feet is cost-effective, quick, and well-tolerated while facilitating the use of the focal zone advantage to assess for FBs. The dynamic nature of bedside ultrasound elevates its overall clinical utility with minimal contraindications or limitations when compared to X-ray, CT, or MRI. Providers in emergency departments, primary and urgent care settings can utilize the water bath and the focal zone advantage presented in this technical report to advance their primary or subsequent assessments for FBs in the presence of skin and soft tissue pain, inflammation, or infection.
